# Animals on the Stage—And off

**Published:** 1901-05-25

**Authors:** 


### EDITOR'S LETTER-BOX.

[Our Correspondents are reminded that prolixity is a great bar to publication, and that brevity of style and conciseness of statement greatly facilitate early insertion.]

### ANIMALS ON THE STAGE—AND OFF.

To the Editor of *The Hospital*.

MR. BENJN. BRYAN, of the Royal Society for the Prevention of Cruelty to Animals, writes:—

"Sir,—I am directed by Mr. Colam to state that having read the article in your issue of the 27th inst. headed "Animals On the Stage—and Off," he desires to inform you that the statements based on the authority of Mr. Bensusan are not new, and have been fully answered by him. In proof of this I am to send you a copy of *The Animal World* for December, 1899, at p. 178 of which you will find an article on the subject. From this you will see that as Mr. Bensusan would give no assistance the society made efforts on its own behalf to obtain information, but did not succeed. Had it done so there would have been no delay in taking proceedings. Please make the necessary explanation to your readers."

[With the above letter Mr. Bryan enclosed a copy of *The Animal World* for December, 1899, containing an article entitled "Mr. Bensusan's Extraordinary Allegation," from which we gather that several years ago Mr. Bensusan had published in another paper certain allegations respecting most horrible cruelty being perpetrated by trainers of animals in London underground cellars, and had reproached the society for failing to prosecute the offenders; that an application had been promptly made to Mr. Bensusan to give the addresses of the cellars, the names of the offenders, or any other particulars by which an investigation could be commenced; that Mr. Bensusan had declined to supply any such information; that from his refusal to give any help to the society, its officers were unable to verify or disprove his allegations; and that notwithstanding the representations made to him by the representative of the society "the accuser remained obdurate and refused to give the least clue."

We also learn, in the words of the writer in *The Animal World*, that after a lapse of three years Mr. Bensusan "renewed these allegations and reproaches, but with a notable extraordinary addition. He now says: 'I once gave to Mr. John Colam the names of some of the worst offenders.'" The truth of this statement the writer in *The Animal World* absolutely denies. "On the contrary," he says, "although repeatedly pressed, Mr. Bensusan emphatically declined to give a single name, a single address, or a single clue to Mr. John Colam."

The article goes on to say, "At the time six detective officers were told off to discover the alleged dens of iniquity. Not a particle of evidence was found by those detectives in support of the original statements, though the inquiry lasted several weeks; nor by the special commissioner appointed by the *Daily Chronicle* to discover the offenders, though that commissioner apparently had interviews with Mr. Bensusan, and with some other person in alliance with Mr. Bensusan. Neither person would reveal a tittle of evidence leading to the detection of the cruelty referred to, which stood then, and still stands, on their unsupported accusation." Mr. Bryan has also favoured us with an extract from the annual report of the society for the year 1896, to show how long this accusation has been going on, and to assure us that the society have made proper efforts to probe their veracity.—ED. "THE HOSPITAL."]

